# Distinguishing vigilance decrement and low task demands from mind‐wandering: A machine learning analysis of EEG

**DOI:** 10.1111/ejn.14863

**Published:** 2020-06-28

**Authors:** Christina Yi Jin, Jelmer P. Borst, Marieke K. van Vugt

**Affiliations:** ^1^ Bernoulli Institute for Mathematics, Computer Science and Artificial Intelligence University of Groningen Groningen The Netherlands

**Keywords:** alpha oscillation, independent component analysis, mind‐wandering, support vector machine, task demands, vigilance

## Abstract

Mind‐wandering is a ubiquitous mental phenomenon that is defined as self‐generated thought irrelevant to the ongoing task. Mind‐wandering tends to occur when people are in a low‐vigilance state or when they are performing a very easy task. In the current study, we investigated whether mind‐wandering is completely dependent on vigilance and current task demands, or whether it is an independent phenomenon. To this end, we trained support vector machine (SVM) classifiers on EEG data in conditions of low and high vigilance, as well as under conditions of low and high task demands, and subsequently tested those classifiers on participants' self‐reported mind‐wandering. Participants' momentary mental state was measured by means of intermittent thought probes in which they reported on their current mental state. The results showed that neither the vigilance classifier nor the task demands classifier could predict mind‐wandering above‐chance level, while a classifier trained on self‐reports of mind‐wandering was able to do so. This suggests that mind‐wandering is a mental state different from low vigilance or performing tasks with low demands—both which could be discriminated from the EEG above chance. Furthermore, we used dipole fitting to source‐localize the neural correlates of the most import features in each of the three classifiers, indeed finding a few distinct neural structures between the three phenomena. Our study demonstrates the value of machine‐learning classifiers in unveiling patterns in neural data and uncovering the associated neural structures by combining it with an EEG source analysis technique.

## Graphical abstract

Mind‐wandering is a different mental phenomenon from both low vigilance and being in a low‐demand task situation (upper panel). Mind‐wandering is associated with activity arising from the left STG (lower panel left bottom corner). Low vigilance is characterized by alpha oscillations arising from the brainstem (lower panel right top corner). Low task demands are associated with activity in a parietal–temporal–occipital network (lower panel left top corner).

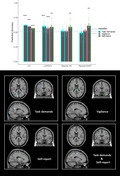

## 1 INTRODUCTION

Mind‐wandering is a ubiquitous mental phenomenon that occurs throughout our daily life in a relatively uncontrolled way. For instance, when you are working on your paper, you suddenly realize that you have been thinking about your plans for the upcoming weekend for a while even without any clues in the environment to trigger such a daydreaming. In psychology, mind‐wandering is commonly defined as self‐generated thought that is irrelevant to the current task and oriented toward other things such as unfinished long‐term goals (Smallwood & Schooler, 2015). Mind‐wandering can be both positive and negative depending on the situation in which it occurs (Smallwood & Schooler, 2015), how difficult it is to disengage from (van Vugt & Broers, 2016) and how frequently the agent has to reorient their attention back to the current task (Allen et al., 2013).

There are various internal and external factors that determine the likelihood of mind‐wandering. Two of the most important are the level of vigilance as an internal factor and task demands as an external factor. Mind‐wandering is more likely to occur when both vigilance and task demands are low. In this paper, we will investigate whether mind‐wandering is fully dependent on those factors, or whether it is a separate phenomenon. To this end, we will examine the relationship between mind‐wandering, vigilance and task demands through training EEG classifiers to do across‐categorization predictions. If classifiers trained on task demands and/or vigilance levels cannot predict the occurrence of mind‐wandering, we can conclude that mind‐wandering is indeed a distinct phenomenon. We decided to use machine‐learning classifiers instead of standard statistical analyses that rely on averages, as those approaches can be fooled by an apparent average overlap that is not accompanied by precise relationships on individual trials. Classifiers are in that respect much more precise, because for a classifier to generalize from one task or context to the next, the relationships need to be present on individual trials.

In the remainder of the introduction, we will review the connections between mind‐wandering, vigilance decrement and the effects of task demand.

### 1.1 Mind‐wandering and vigilance decrement

Mind‐wandering is known to depend on vigilance: As a person's vigilance decreases, their mind‐wandering tends to increase (Krimsky, Forster, Llabre, & Jha, 2017). A vigilance decrement is defined as reduced alertness to critical events (Haubert et al., 2018). The difference between mind‐wandering and vigilance decrement is hard to tell by behavioral studies, since both are associated with worse task performance due to a withdrawal of attentional resources from the main task. For example, Pattyn, Neyt, Henderickx, and Soetens (2008) showed that vigilance decrement was accompanied by an increasing error rate. Also during mind‐wandering people tend to make more mistakes (McVay & Kane, 2013).

In neuroimaging research, reduced vigilance is found to be associated with increased activity in the default mode network (DMN), which might play a role in the reduction in the ability to control responding that has been observed with reduced vigilance (Bogler, Vowinkel, Zhutovsky, & Haynes, 2017). Interestingly, the DMN is also regarded as a key network underlying mind‐wandering (Bonnelle et al., 2011; Christoff, Irving, Fox, Spreng, & Andrews‐Hanna, 2016; Durantin, Dehais, & Delorme, 2015). Additionally, electroencephalography (EEG) studies have shown that vigilance declines are accompanied by a reduced P3b (Haubert et al., 2018). P3b is an event‐related potential (ERP) observed as a positive deflection peaking at around 500 ms after stimulus onset with a maximal distribution over the parietal area. It is thought to reflect the amount of mental resources devoted to the primary task (Polich, 2007). Similar P3 reductions were found in people reporting to be mind‐wandering (Barron, Riby, Greer, & Smallwood, 2011; Smallwood, Beach, Schooler, & Handy, 2008).

Together, this demonstrates that vigilance decrements and mind‐wandering overlap in their consequences for behavior and brain activity measured through EEG or functional neuroimaging. This suggests that either the two are different facets of the same phenomenon, or alternatively, that they are two phenomena co‐occurring with each other. Regardless, the evidence discussed above suggests that mind‐wandering will occur when people have reduced vigilance.

Interestingly, there is other evidence suggesting that mind‐wandering is not necessarily linked to performance deficits resulting from decreased vigilance (Neigel, Claypoole, Fraulini, Waldfogle, & Szalma, 2019). For example, while sleep deprivation is a clear cause of fatigue and lower vigilance (Massar, Lim, Sasmita, & Chee, 2019), sleepiness and mind‐wandering have been shown to each have independent effects on task performance (Stawarczyk & D'Argembeau, 2016).

One goal of the current study is to address this controversy about the relationships between mind‐wandering and vigilance. Our hypothesis starts from the aforementioned “vigilance decrement == mind‐wandering” assumption, according to which a vigilance decrement could be an indication of mind‐wandering. We have previously demonstrated that it is possible to detect the occurrence of mind‐wandering on a single‐trial level with a machine‐learning classifier (Jin, Borst, & van Vugt, 2019). Such classifiers can also be trained to detect other mental states, including potentially vigilance. If mind‐wandering and vigilance decreases are both manifestations of the same underlying process, then a classifier that can successfully detect low‐vigilance moments should be able to detect the occurrence of mind‐wandering as well.

### 1.2 Mind‐wandering and task demands

Another factor that affects the occurrence of mind‐wandering is the level of task demands. Behavioral studies have demonstrated that increasing task demands tend to reduce the mind‐wandering rate, provided that task demands do not exceed the capacity of the participants (Randall, Beier, & Villado, 2019; Smallwood et al., 2011). Interestingly, neuroscience studies show that mind‐wandering relies on similar neural structures as being on task; both are associated with the activation of visual cortex and the prefrontal cortex—which is one of the main reasons why it is difficult to distinguish mind‐wandering from other mental processes (Christoff, Ream, & Gabrieli, 2004). In addition, recent evidence shows that the DMN is involved in both on‐task and off‐task processing (Sormaz et al., 2018). These overlapping neural structures can be explained by the fact that both successful task performance and the generation of mind‐wandering episodes require the involvement of executive attention (Smallwood & Schooler, 2006). A decline in task performance resulting from mind‐wandering could be viewed as the result of the mind‐wandering process “winning” the competition for cognitive resources from the main task. If the occurrence of mind‐wandering depends on a competition with task goals, then increasing the importance of these goals should also decrease the amount of mind‐wandering because there are fewer cognitive resources left to support mind‐wandering (van Vugt, Taatgen, Sackur, & Bastian, 2015). If this is true, then low levels of task demands should be accompanied by high levels of mind‐wandering, while high levels of task demands should be accompanied by low levels of mind‐wandering.

The goal competition account gives rise to the second hypothesis we address in the current study. Following the same logic as in the “vigilance” section, if the “low demands == mind‐wandering” assumption is true, a trained classifier detecting people's attentional state when performing tasks of low versus high demands should be able to detect mind‐wandering as well. That is, low task demands should predict a higher likelihood of mind‐wandering, while high task demands are expected to be associated with more “on‐task” responses.

### 1.3 Alpha oscillation as a neural marker

While most of the previously discussed studies of mind‐wandering relied on neuroimaging or event‐related potentials, there have been also several studies using oscillatory EEG information. Oscillatory EEG studies have demonstrated that vigilance, mind‐wandering and task demands are all sensitive to periodic activity in the 8–12 Hz alpha range.

In vigilance research, alpha oscillations are regarded as an index of alertness. When people enter a calm and relaxed state, alpha oscillations tend to increase in amplitude to the extent that it is often even visible to the naked eye. Reduced vigilance is shown to be accompanied by increased alpha power (Molina, Sanabria, Jung, & Correa, 2017). In a simulated driving task, the participants had increased alpha band activity as they were fatigued (Craig, Tran, Wijesuriya, & Nguyen, 2012).

In research on attention, task performance on a perceptual task was shown to be better when alpha power, especially at posterior sites, was observed to be lower (Hanslmayr et al., 2005). Alpha power has also been shown to be lower for detected compared to undetected stimuli (Ergenoglu et al., 2004), predictive of visual discrimination performance (van Dijk, Schoffelen, Oostenveld, & Jensen, 2008) and lower contralateral to the attended visual hemisphere (Thut, Nietzel, Brandt, & Pascual‐Leone, 2006). In that sense, alpha power could index the deployment of mental effort. If people devote more effort or resources to the external task, their alpha oscillatory activity at the parietal–occipital sites is expected to be inhibited.

In mind‐wandering research, alpha oscillations have been shown to be a consistent predictor of this mental state (Baldwin et al., 2017; Jin et al., 2019; Macdonald, Mathan, & Yeung, 2011). In a recent study, we verified this idea by contrasting the ability of different EEG features to predict mind‐wandering. Oscillatory power in the alpha band from occipital channels turned out to be the most predictive EEG feature for mind‐wandering (Jin et al., 2019). This result is compatible with findings from neurofeedback research, which show that a reduction in alpha oscillatory power is accompanied by an increase in people's focus level in a post‐training task (Ros et al., 2013).

The primary role attributed to alpha oscillations is attentional suppression: Alpha power enhancement allows the participant to ignore irrelevant stimuli more effectively (Kelly, Lalor, Reilly, & Foxe, 2006) and is thought to reflect suppression of the visual dorsal stream during the construction of internal operations (Tuladhar et al., 2007). In that sense, alpha oscillations might function to prevent the attentional resources from being captured by “unwanted” outside stimuli, thereby keeping the internal operations from being interfered with. The top‐down modulation of attention during the early encoding phase has been reported to be supported by the prefrontal cortex (PFC). Contributing to the selectivity of the attention when resources are guided toward the relevant stimuli and ignoring the distractions, this top‐down modulation is found to be associated with frontal–parietal coherence in the alpha band (Zanto, Rubens, Thangavel, & Gazzaley, 2011).

### 1.4 Current study

Based on the similarity of reported alpha oscillation activity between mind‐wandering versus on‐task, low versus high vigilance, and low versus high task demands, combined with our previous two assumptions (“mind‐wandering == low vigilance,” “mind‐wandering == low task demands”), we hypothesized that a successful classifier based on alpha oscillations that is trained on a vigilance or task demand categorization should be able to detect mind‐wandering as well.

To test this idea, we designed a visual search task in which we manipulated the levels of task demands. The main stimuli were visual search panels with geometric graphics. In the low‐demand condition, participants passively viewed the search panel and hit a key when done. In the high‐demand condition, participants counted a specific target during the presentation of the search panel and entered their count on the keyboard. The reason for employing such a paradigm is to ensure that participants receive the same input to their perceptual system while at the same time having to devote varying amounts of mental effort between conditions. Therefore, if we find any difference in their brain signal during sensory processing, we can rule out the possibility that it was caused by the different stimuli or response key presses per se. Crucially, participants were intermittently probed to provide a subjectively rated estimate of the extent to which they were currently mind‐wandering or on‐task.

After the visual search task, participants performed a sustained‐attention‐to‐response task (SART), which is a classic paradigm used to study mind‐wandering. In this task, participants are presented with a frequent GO stimulus requiring a response and an unpredictable rare NOGO stimulus, which is used to detect momentary lapses of attention since participants have to overcome the automatic tendency to respond using inhibitory control. The same thought probe questions as used for the visual search task were inserted into the trial sequence of the SART.

In our machine‐learning approach, the training data always came from the visual search task. Based on this, three models could be built: a vigilance classifier, a task‐demand classifier and a self‐rated mental state classifier. The test data always came from the other task, SART, and were only labeled by the self‐reported attentional state. If our hypothesis is correct, the classifier trained on the vigilance labeling or task demands labeling should achieve an equivalent level of prediction accuracy as the classifier trained on mind‐wandering in predicting mind‐wandering in the SART. If not, or in other words, if the vigilance and/or task demands classifier score lower than the self‐report classifier, this would indicate that mind‐wandering is a different mental state from low vigilance or being in a task with low demands as indicated by different EEG patterns.

The goal of the current research is to determine whether vigilance decrement and low task demands are similar to mind‐wandering, or whether these are qualitatively different processes. We aim to achieve this goal through detecting the relevant EEG patterns using machine learning. If mind‐wandering is shown to be different from the other two mental phenomena, this allows us to examine how the classifiers differentiate between them. The results will help us to further understand the commonalities and distinctions between mind‐wandering, vigilance decrement and performing a task with low demands while they all show a similar “attentional decline” behaviorally.

## 2 METHODS

### 2.1 Participants

Thirty participants (16 females, age 18–31 years, *M* = 23.73, *SD* = 3.47) took part in the current study. They reported normal or corrected‐to‐normal vision. The research was conducted in accord with the Declaration of Helsinki and approved by the Research Ethics Committee of the Faculty of Arts (CETO), University of Groningen. Participants gave written informed consent. They were paid 16 euros for their participation in the two‐hour experiment (duration includes the EEG setup time). They were debriefed with the main goal of the task after the experiment.

### 2.2 Experimental procedure

The experiment consisted of two tasks: a visual search task and a SART. Participants performed the experiment in a sound‐attenuated booth. Both tasks were programmed and presented in the PsychoPy3 standalone version (Peirce et al., 2019). Continuous EEG was recorded during the task with a Biosemi 32‐channel system.

**FIGURE 1:**
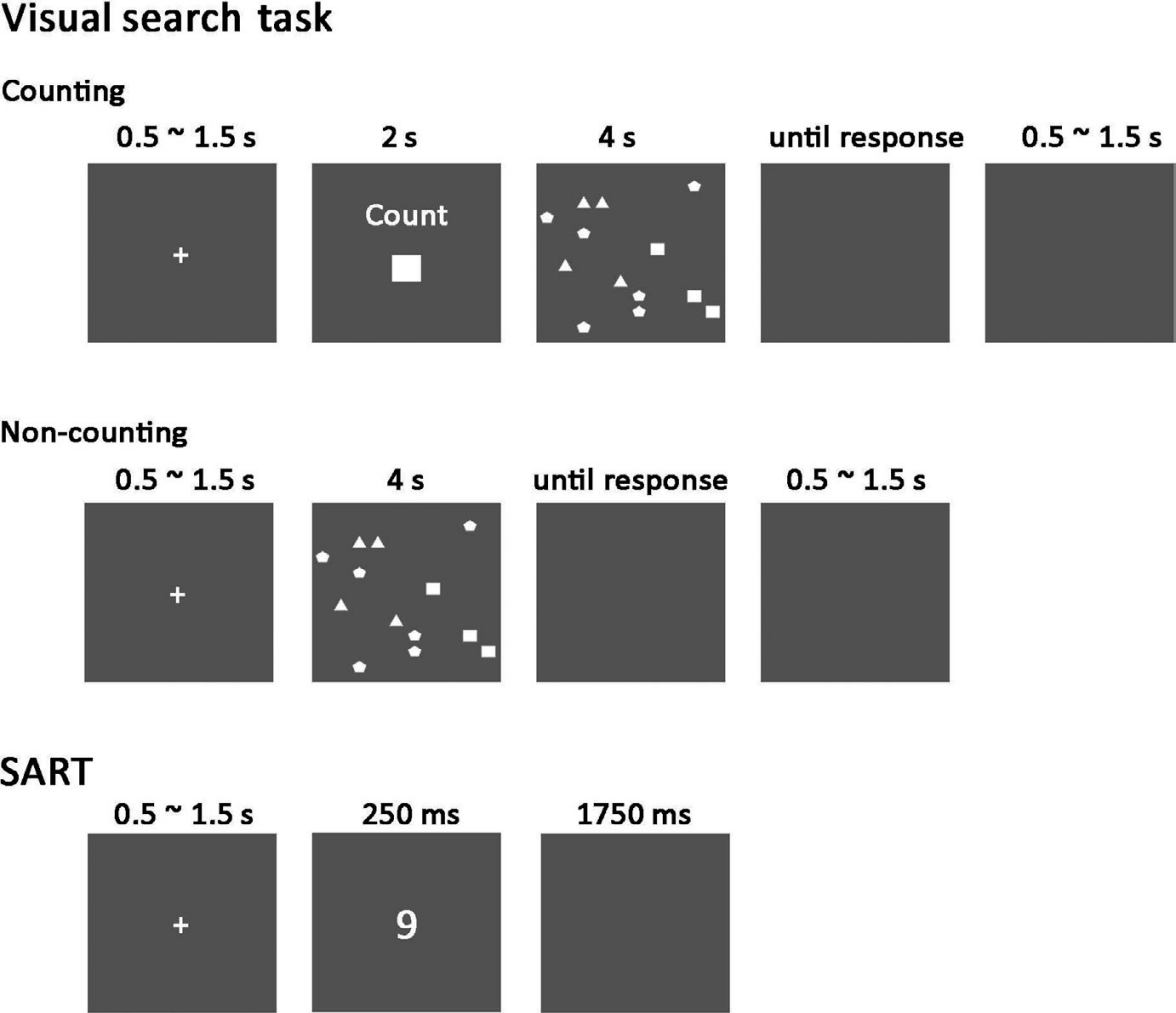
Trial sequences of the visual search task (top) and the sustained‐attention‐to‐response task (SART; bottom). In the counting visual search task (difficult task condition), participants were required to count the number of occurrences of the target stimulus in the search panel and press the number key indicating the counting result as a response. In the non‐counting visual search task (easy task condition), participants were asked to passively view the search panel and simply press “j” after its termination. In the SART, participants were required to press “j” every time they saw a non‐three digit and to withhold their response when the digit “3” occurred

The visual search task involved two conditions that manipulated task demands: a counting (high demand) and a non‐counting (low demand) condition. In both conditions, a display with geometric shapes was shown to the participants (Figure 1). Task conditions were varied on a block‐by‐block basis. Each block started with the instruction for the current block. In a counting block, the instruction was “Count the targets and press the number to indicate the answer after the display disappears.” In the non‐counting block, the instruction was “Press J after the display disappears.” The experiment was divided into four sessions. Each session consisted of five blocks of the same condition. The session sequence followed either an “A‐B‐A‐B” or “B‐A‐B‐A” style, which were balanced between participants. There were in total 10 blocks for each condition.

All stimuli were shown in white against a gray background (Figure 1; PsychoPy3 default). Each trial started with a fixation cross shown for a random duration sampled from the interval between 0.5 and 1.5 s. After the fixation cross disappeared, participants saw a trial instruction for 2 s about what target to count in the following search panel in the counting condition. A target could be a triangle, square or pentagon. Following the instruction, a display consisting of geometric graphics was presented. Participants were asked to count the specified target or just passively view it. The search panel lasted for 4 s. After that, there was a blank screen during which participants had infinite time to make a response: In the counting task, they were required to input a number on the numeric keypad to indicate their counting result; in the non‐counting task, they simply pressed “j.” The inter‐trial‐interval was on average 1 s (range 0.5–1.5 s).

The search panel was a centrally localized 10.6 × 10.6 cm square (visual angle 5.0° × 5.0°), equally divided over an invisible 10 × 10 grid (100 stimulus holders). Each geometric graphic was randomly assigned to one holder. The number of copies of each shape varied between two and eight in each search panel. The target shape was counterbalanced between trials. There were 21 trials in each block, where each block lasted for approximately 3 min.

As in previous mind‐wandering studies, we examined participants' mental states by means of thought probes. Unlike previous studies, however, the thought probes were not randomly interspersed between trials but rather were placed at the end of each block. In each thought probe, participants were shown a continuous rating scale, asking “To what degree were you focusing on the task just now?”. Participants could choose an integer between −5 and 5 to indicate their momentary attentional level, with anchors “−5” for “totally mind‐wandering,” “−2” for “mind‐wandering,” “0” for “uncertain,” “2” for “focused” and “5” for “highly focused.”

The SART is a GO‐NOGO‐based experiment paradigm, in which participants are supposed to press a button when they encounter GO stimuli (which are presented most of time) and withhold their response to occasionally presented NOGO stimuli. Here, the main stimuli in the SART were single digits from 1 to 9. Participants were required to press “j” as soon as they saw the number with an exception for “3” (around 11% chance), which was the NOGO stimulus. Numbers were counterbalanced across trials. Each trial started with a cross fixation lasting for 0.5–1.5 s, followed by a digit for 250 ms and a blank screen for 1,750 ms. Participants could press the button any time after the digit stimuli onset. The SART consisted of 12 blocks with block length varied between 2 and 7 repetitions of the 9 digits (18–63 trials, approximately for 1–3 min). At the end of each block, they were asked to rate their momentary attentional level on the same scale used in the visual search task.

Participants were seated at a distance of approximately 60 cm from the display. Before the experiment, they were told the aim of the experiment was to study people's attentional fluctuations. They were further explained about the rating scale they would encounter during the tasks and were asked to use this rating scale honestly and to indicate the content of their thoughts seriously. Participants performed practice trials until they reached a certain level of accuracy (for the visual search task, 4 out of 5 correct responses in the most recent 5 consecutive trials; for the SART, one correct withholding of their response to the NOGO stimulus “3”; on average, participants practiced 11.8 ± 2.3 trials for the visual search task and 14.7 ± 9.3 trials for the SART) before they performed the real experiment. Accuracy feedback was given during the practice to facilitate learning of the task but withheld during the actual experiment. After finishing each block, participants could take a short break if they so desired.

### 2.3 Trial classification

The training of classifiers to detect mind‐wandering, vigilance and task demands is based on three different ways of labeling the data. The task demands classifier labels are based on the experimental conditions, in which counting task trials reflect high task demands and non‐counting task trials reflect low task demands. The vigilance classifier labels are based on the time course of the task, which categorizes the first half of the trials, during which the participant is still relatively fresh, into the high‐vigilance category, and the second half of the trials into the low‐vigilance category. The mental state classifier labels were based on the self‐rated attentional level, which labels the three trials preceding each probe (comprising a total duration of approximately 24 s^1^) as mind‐wandering if the rating is smaller than 0, and as on‐task if the rating is larger than 0 (thus, ratings were converted into a binary classification of on‐task vs. mind‐wandering; ratings of 0 were removed because they indicated “uncertain”; this removed 9.6% of the ratings).

Data of the SART were only classified as either mind‐wandering or on‐task based on the participant's self‐report ratings of their mental state—and not in terms of vigilance or task difficulty.

### 2.4 Behavioral data analysis

Individual accuracy was computed and a group‐level comparison within each categorization was performed using paired *t* tests. Individual mean response time (RT) was computed on the basis of correct trials only, additionally excluding 2.5% of the data at each end of the RT distribution to exclude outliers. Due to the non‐normality, RT was log‐transformed before being compared with paired *t* tests.^2^ The effect size was reported as Cohen's *d*.

Individual focus‐level rating scores were computed excluding ratings of “zero,” which indicated the participant was “uncertain” about their mental state. A group‐level one‐factor repeated‐measures ANOVA was performed to examine the differences between the counting, non‐counting and SART. When we observed a significant difference, pairwise comparisons were performed using paired *t* tests and reported with adjusted *p*‐values using the Bonferroni correction for multiple comparisons. Meanwhile, the focus‐level rating scores were also examined using a group‐level *t* test between the first half (“high vigilance”) and the second half (“low vigilance”) of the visual search task.

### 2.5 EEG recording and offline preprocessing

EEG was recorded by a Biosemi 32‐channel system with six additional electrodes to detect eye movements and mastoid signals. The scalp channel locations are within the International 10–20 System. The sampling rate was 512 Hz. An electrode next to the vertex electrode was used as the reference during recording. Impedances were kept below 40 kΩ. The Biosemi hardware does not have any high‐pass filtering. An anti‐aliasing filtering (low‐pass filtering) is performed in the ADC's decimal filter (hardware bandwidth limit), which has a fifth‐order cascaded integrator‐comb filter response with a ‐3 dB point at 1/5th of the selected sample rate. For more, see the Biosemi website (https://www.biosemi.com/faq/adjust_filter.htm)

Offline EEG preprocessing was performed using the EEGLAB toolbox (v14.1.1, Delorme & Makeig, 2004) on MATLAB (v2013b). Bad channels were defined as channels with excessive spikes or obviously noisier than surrounding channels. There were two participants with bad channels—each having exactly one bad channel. Data for each of these two bad channels were replaced through spherical interpolation based on the neighboring electrodes. Continuous data were re‐referenced to the average signal of both mastoids, band‐pass‐filtered (0.1–42 Hz), down‐sampled to 256 Hz and segmented to epochs of 4 s (1 s before and 3 s after stimulus onset). Baseline correction was performed using the interval of −200 to 0 ms as a baseline. Data were visually inspected for large movement artifacts. An infomax independent component analysis (ICA) was used to detect and remove ocular artifacts. EEG epochs were visually inspected again after the ICA artifact removal to ensure the remaining signal to be clean.

**FIGURE 2:**
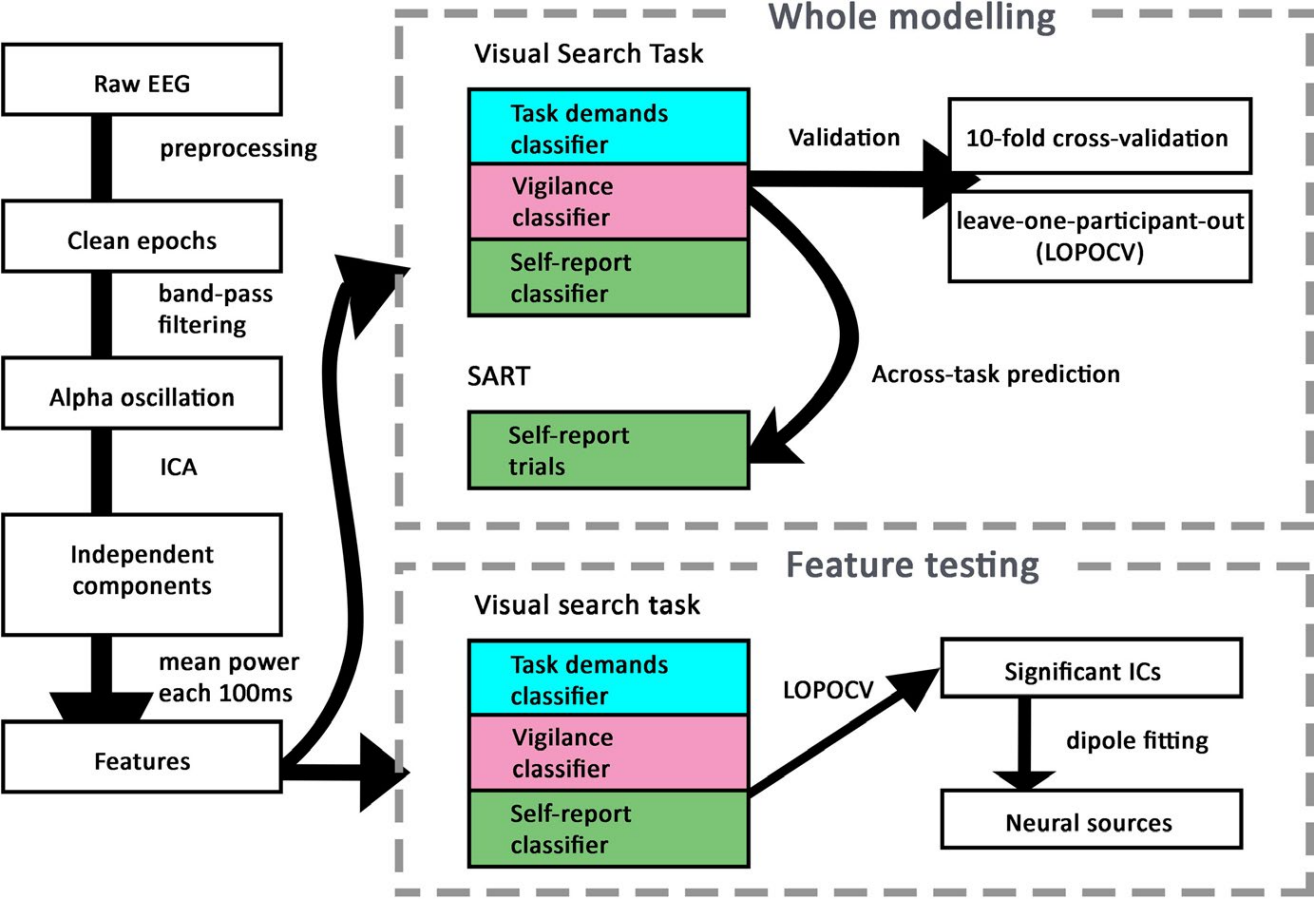
EEG data analysis pipeline. After preprocessing, we performed band‐pass filtering on the clean EEG signals to obtain the alpha oscillatory activity (8.5–12 Hz). A further independent component analysis (ICA) was performed on the resulting alpha power over all the participants to isolate the time series into 32 independent components (ICs), of which we took 100 ms as a bin and computed the mean power over each bin as the final features for classification. The classifier training consisted of two stages. First, we used the power of all 32 ICs as features and trained three types of the classifiers—task demands classifier, vigilance classifier and self‐report classifier—using the data from the visual search task (whole modeling). All the classifiers were validated through both the 10‐fold cross‐validation and the leave‐one‐participant‐out cross‐validation (LOPOCV). For the across‐task predictions, we tested all the three classifiers on the data from the SART, which were only labeled based on the self‐reports. Secondly, we took the power of each IC to train the three classifiers, respectively (feature testing). The performance of each IC during the LOPOCV was compared to the chance level using the one‐sample *t* tests to obtain the significant ICs in each type of classifier. Further, we applied the dipole‐fitting analysis to each of the significant ICs to unveil the underlying neural correlates [Colour figure can be viewed at wileyonlinelibrary.com]

An EEG data analysis pipeline including preprocessing, feature extraction, machine learning and feature testing procedures is illustrated in Figure 2.

### 2.6 Feature computation

The classifier features consisted of the time window from 600 ms before to 600 ms after stimulus onset (stimulus onset is defined as the search panel onset in the visual search task and the digit onset in the SART).^3^ In these windows, we computed alpha band activity based on a frequency range of 8.5–12 Hz. The ideal filter kernel was in a “plateau” shape in which 8.5–12 Hz were set as 1, while the other frequencies were set as 0 with 20% as transition width. The kernel length was set to at least three cycles of the lowest frequency (8.5 Hz). The kernel was constructed by the MATLAB function *firls()*. The sum of squared errors (*SSE*) of the constructed kernel and the ideal kernel was 0.1. To obtain oscillatory power, the band‐pass‐filtered data were Hilbert‐transformed by means of the MATLAB function *hilbert()*. From this analytical signal (in a complex form), the oscillatory activity was computed through taking the real parts, and power was computed as the square of the vector length at each time point (Cohen, 2014).

Independent component analysis was used to compute features in the current study. The main reason to use such IC‐based features is to allow good fitting of the follow‐up source localization analysis. ICs are assumed to be unmixed signals from different sources (Cohen, 2014) and are recommended in reconstructing the signal source (Delorme, Makeig, Fabre‐Thorpe, & Sejnowski, 2002). IC loadings were obtained by applying ICA on the alpha band oscillations from all participants. Features in our classification analyses are the IC activities, which can be regarded as a weighted sum of signals from each channel using the obtained IC loadings during the ICA. Thus, each IC activity of each trial is a single oscillatory time course signal of which power can be computed. We took a step size of 100 ms and separated the 1,200 ms epoch into 12 bins, for each of which the average power was computed. This resulted in 12 features for each IC and 32 ICs leading to 12 × 32 = 384 features in all.

### 2.7 Classifier training and testing

We used a support vector machine classifier (SVM) due to its excellent performance in EEG classification in previous studies of mind‐wandering (Jin et al., 2019; Lotte, Congedo, Lecuyer, Lamarche, & Arnaldi, 2007). An important reason for using an SVM is that this classifier does not require a linear relationship between the features, which is an advantage in classification when we cannot make a specific assumption about the shape of the interacting pattern between the features. We applied the radial basis function (RBF) kernel during the training to take advantage of the non‐linear power of the SVM. The regularization term C was set to be 1. The parameter γ of the RBF kernel was set to be 1/(data dimension).

The training and testing procedures ware mainly performed by the *e1071* package in R combined with customized code. Each feature was z‐transformed, and the training sample was balanced between the two classes using the random over‐sampling method. The training was performed on the pooled data of the visual search task from all participants and validated in a combined 10‐fold cross‐validation (CV) and a leave‐one‐participant‐out cross‐validation (LOPOCV) framework. In each iteration, one participants' data were kept aside for validation purposes, while the remaining data constituted the training sample, on which a classifier was built and validated by means of 10‐fold CV. Based on the different categorizations as described in Section 2.3, we trained three classifiers in each iteration: a task‐demand classifier, a vigilance classifier and a self‐reported mental state classifier (to assess mind‐wandering).

Furthermore, the classifiers were tested in an across‐task fashion (Jin et al., 2019) to predict the data of the SART. We expected that the task demand classifier or the vigilance classifier could achieve an equivalent level of accuracy to the self‐reported mental state classifier in predicting being on‐ versus off‐task in the SART if task demands and vigilance are comparable mental states to mind‐wandering. The theoretical chance level of a binary classification problem is 0.5. However, we decided to use a corrected chance level considering the data size according to Combrisson and Jerbi (2015). At the conventional significance threshold of *p* < 0.05, the corrected chance level is 50.77% for the task demands/vigilance classifiers (both *n* = 11,436) and 52.14% for the self‐report classifiers (*n* = 1,494). The chance levels in the later sections of the manuscript all refer to these corrected chance levels.

### 2.8 Feature testing and source localization

To investigate the possible neural interpretation of the trained classifier, we did a feature testing analysis, for which each IC was used as the only predictor in training the classifier. The predictiveness of each feature (IC) was quantified by the accuracy during the LOPOCV. For each feature, we could then determine whether it contributed significantly to the prediction of interest by comparing its accuracy to the chance level using one‐sample tests. A further dipole‐fitting procedure was performed on those significant ICs to unveil the underlying neural structure. The dipole fitting and visualization were performed using the DIPFIT2 plug‐in of EEGLAB. A boundary element model (BEM) was used to fit the data. As our electrode locations are all within the International 10–20 System, we simply used the standard channel coordinates associated with the head model. Dipole positions were reported in Montreal Neurological Institute (MNI) coordinates.

## 3 RESULTS

### 3.1 Behavioral results

**FIGURE 3:**
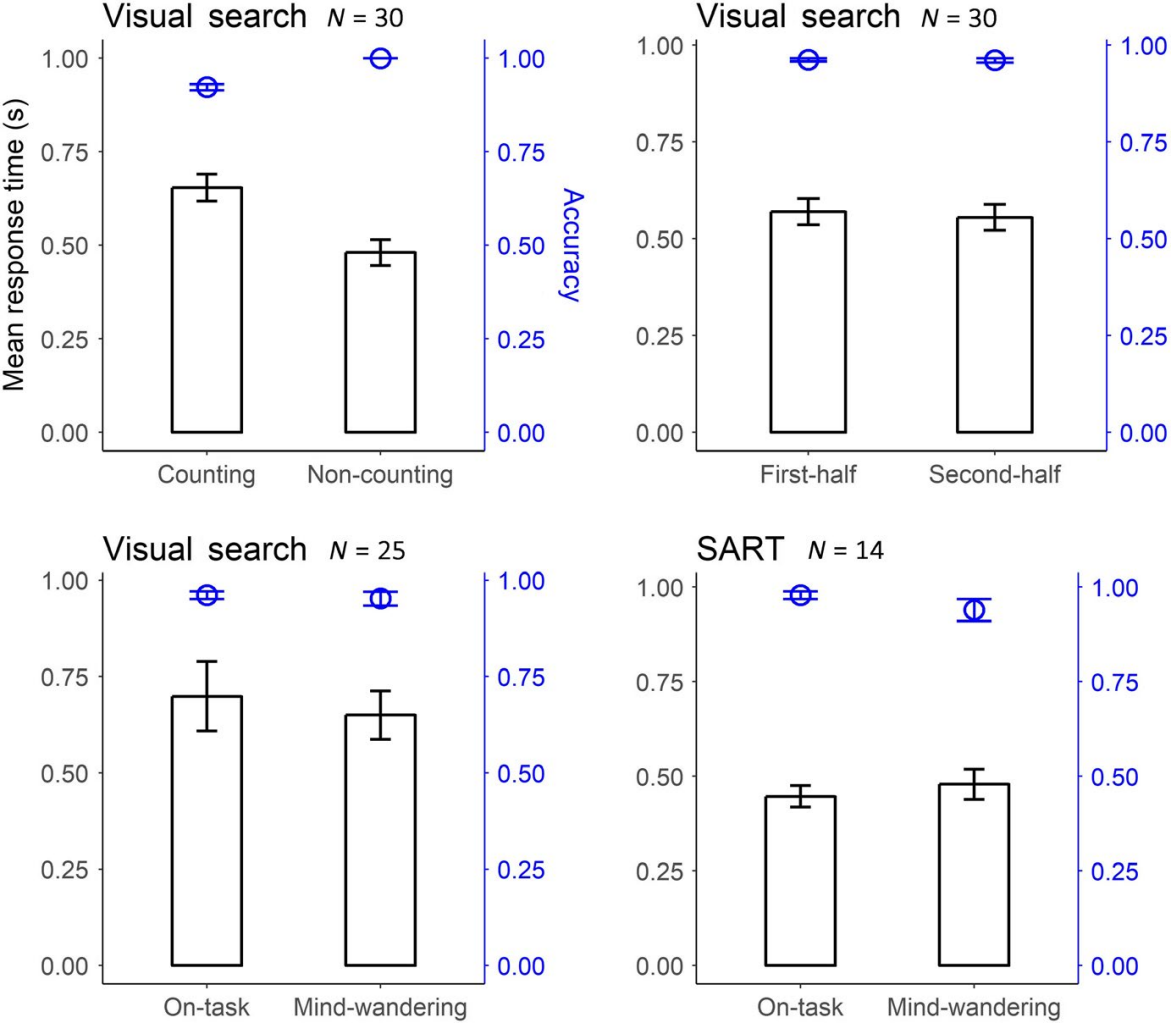
Response time (in seconds) and accuracy for conditions differing in task demands (top left), vigilance (top right) and self‐reported mental state (bottom). Response time is shown bars, and accuracy is shown as points. Both error bars indicate one between‐subject standard error (*SE*) [Colour figure can be viewed at wileyonlinelibrary.com]

Figure 3 shows the average accuracy and response time. Paired *t* tests were performed to compare the data between the two conditions. To assess the effectiveness of our task manipulation, we compared counting to non‐counting task performance (our manipulation of task demands). As we intended, there was a significant difference between the two tasks in both the accuracy (*t*(29) = −9.54, *p* < 0.001, *d* = 1.74) and the log‐transformed response time (*t*(29) = 7.16, *p* < 0.001, *d* = 1.31). Participants obtained lower accuracy and made slower responses in the counting task (accuracy: *M* = 0.92, response time: *M* = 0.654 s) compared to the non‐counting task (accuracy: *M* = 1.00, response time: *M* = 0.480 s).

For the comparison between the first and second half of the experiment (our proxy for vigilance), we found no significant difference in the accuracy (*t*(29) = 0.33, *p* = 0.742) but a significant difference in the log‐transformed response time (*t*(29) = 2.23, *p* = 0.034, *d* = 0.41). Participants made faster responses in the second half (*M* = 0.555) compared to the first half (*M* = 0.569 s) of the visual search task.

Because only 25 out of the 30 participants reported mind‐wandering moments (defined as ratings < 0) to the thought probe in the visual search task, the difference between the on‐task and mind‐wandering state could only be compared for these 25 participants (the exact trial count in each condition for each participant can be found in Appendix S2). We did not find any significant differences in task performance between the two self‐reported mental states (mind‐wandering vs. on‐task); neither in accuracy (*t*(24) = 0.53, *p* = 0.597) nor in log‐transformed response time (*t*(24) = −0.04, *p* = 0.966). A Bayes factor analysis of these non‐significant differences suggests that accuracy is equivalent between mind‐wandering and on‐task (BF_10_ is 0.24, indicating that the null hypothesis is more than 4 times as likely as the alternative hypothesis) and the same applies to the log‐transformed response time (BF_10_ is 0.21, indicating that the null hypothesis is almost 5 times as likely as the alternative hypothesis).

Similarly, as there were only 14 participants for whom the reports contained both mind‐wandering and on‐task cases in the SART, we performed paired *t* tests on the data from those 14 participants to examine the effect of the self‐reported mental state on SART performance. The results did not show any significant difference between mind‐wandering and on‐task; neither in accuracy (*t*(13) = 1.31, *p* = 0.214), nor in log‐transformed response time (*t*(13) = −1.25, *p* = 0.235). A Bayes factor analysis shows that this failure to find a significant difference is the result from too much uncertainty in the data to draw any definitive conclusion (Bayes factor BF_10_ is 0.55 for accuracy and 0.52 for log‐transformed response time, in both cases suggesting that the null hypothesis is about twice as likely as the alternative hypothesis).

**FIGURE 4:**
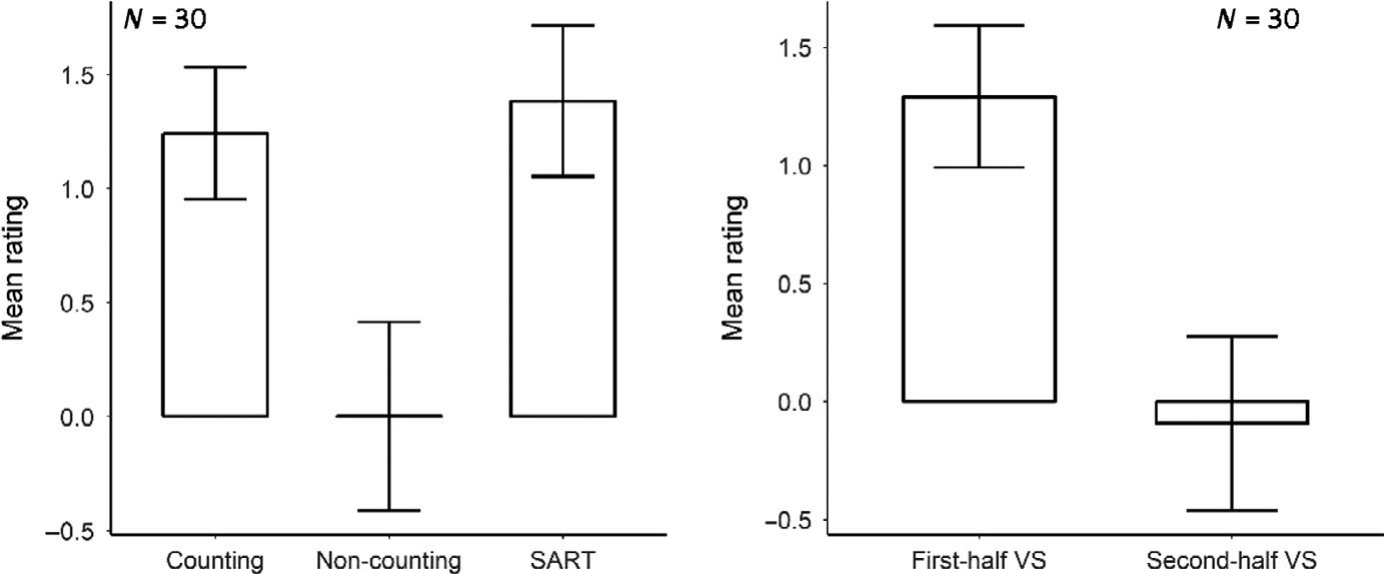
Mean focus‐level rating scores across tasks (left) and across time (right). Error bar indicates one between‐subject standard error (*SE*). SART, sustained‐attention‐to‐response task; VS, visual search task

The averaged focus‐level rating scores were computed excluding ratings of “zero,” which indicated the participant was “uncertain” about their mental state. A group‐level repeated‐measures ANOVA with the factor “task” was performed to examine the differences between the counting, non‐counting and SART, which turned out to be significant (*F*(2, 58) = 11.28, *p* < 0.001, *η* ^2^ = 0.1). Pairwise comparisons using paired *t* tests suggested a significant difference between the counting (*M* = 1.24) and non‐counting tasks (*M* = 0.002, *p*_corrected_ = 0.003) and between the non‐counting task and the SART (*M* = 1.38, *p*_corrected_ = 0.002). Participants rated themselves as being more focused in the counting task and the SART compared to the non‐counting task (Figure 4, left column).

A paired *t* test was performed to examine whether there was a significant difference between the first half (“high vigilance”) and second half (“low vigilance”) of the visual search task. A significant effect of time course was found, indicating that the focus level was higher in the first half (*M* = 1.29) than the second half of the visual search task (*M* = −0.09, *t*(29) = 5.52, *p* < 0.001, *d* = 1.0) (Figure 4, right column).

### 3.2 Classification results

**FIGURE 5:**
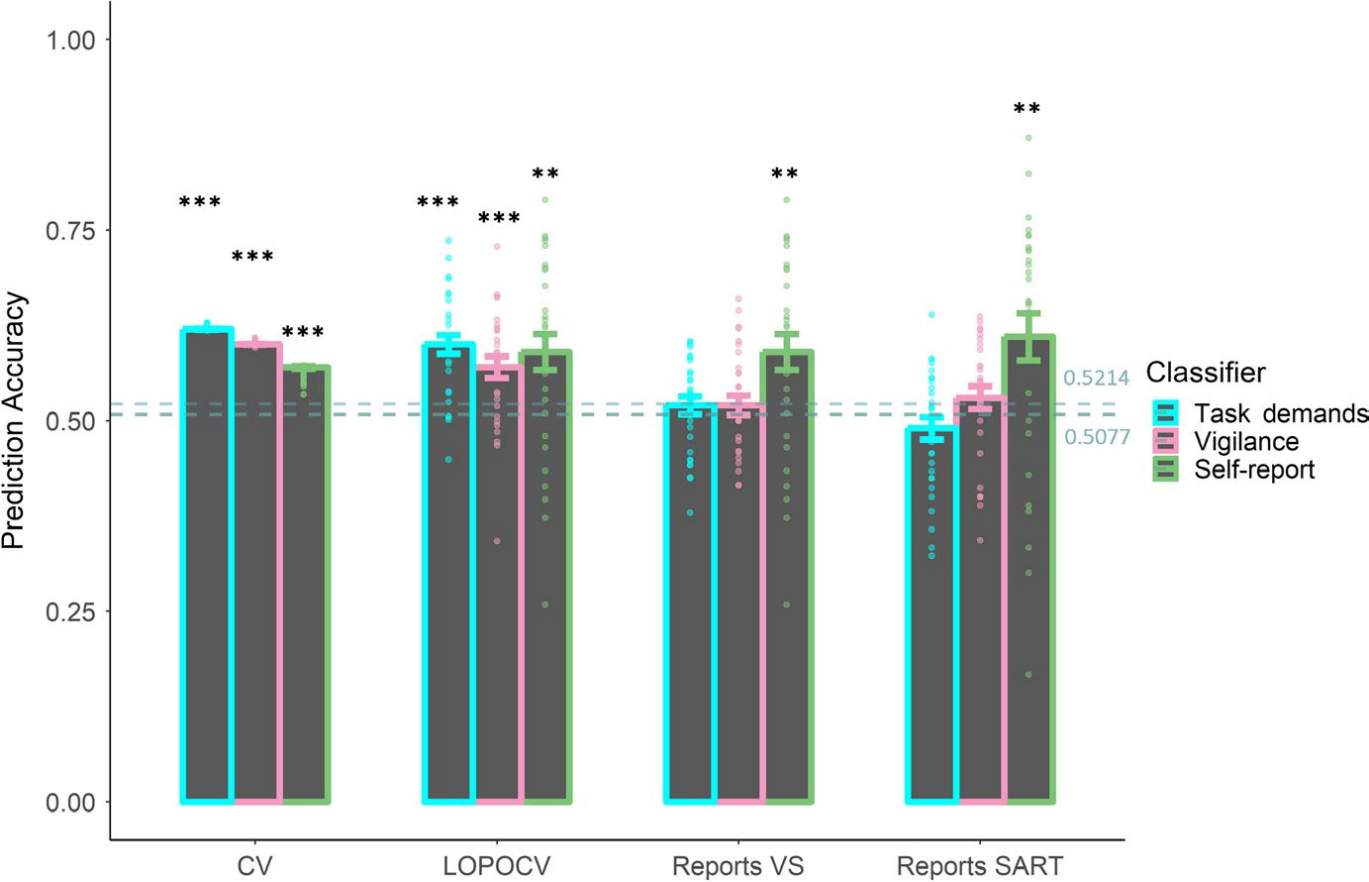
A comparison of performance of the three classifiers in the 10‐fold cross‐validation (CV), the leave‐one‐participant‐out cross‐validation (LOPOCV) and in predicting the self‐reported mental states in both the visual search task (Reports VS) and the SART (Reports SART). Note the self‐report classifier (green bar) is identical in the LOPOCV and Reports VS; the repetitive graphing is to allow for easy visual comparison. The horizontal dashed line indicates chance levels (0.5077 for task demands/vigilance and 0.5214 for self‐report). The error bar reflects one between‐subject standard error (*SE*). Sensitivity and specificity as complementary indicators of classifiers' biased detection can be found in Appendix S3 (An inspection of the sensitivity and specificity of the classifiers indicates that the vigilance/task demands classifiers achieved relatively unbiased detection [comparable sensitivity and specificity] while the self‐report classifier had an obvious biased detection to the on‐task trials [low sensitivity and high specificity]. Similar result has been observed before—the SVM seemed to be good at detecting the majority cases, Jin et al., 2019. However, such biases were not an outcome of the unbalanced dataset since we performed balancing procedure during the training. Instead, it is likely to be caused by the noise during the labeling using the self‐report methodology that increases the difficulty in differentiating mind‐wandering from on‐task, Jin et al., 2019.). Asterisks indicate the difference between the accuracy and chancel level is significant using one‐sample *t* tests (***p < 0.001, **p < 0.01) [Colour figure can be viewed at wileyonlinelibrary.com]

We then examined whether there were systematic differences in brain activity that could be picked up by a classifier. A summary of the different classifiers' performance can be found in Figure 5. *t* tests between the mean classification accuracy and chance level indicated that all the classifiers surpassed chance level in the 10‐fold CV and the LOPOCV analyses (*t*s > 2.80, *p*s < 0.01). However, when predicting the self‐reported mental states in the visual search task or the SART, the task demands classifier and the vigilance classifier remained just around chance level (*t*s < 1.38, *p*s > 0.13); the self‐report classifier showed above‐chance performance in the SART (*t* = 2.89, *p* = 0.007).^4^ In other words, neither the vigilance nor the task‐demand classifiers could predict mind‐wandering while the self‐report classifiers were able to do so across tasks.

### 3.3 Feature testing and dipole fitting

**FIGURE 6:**
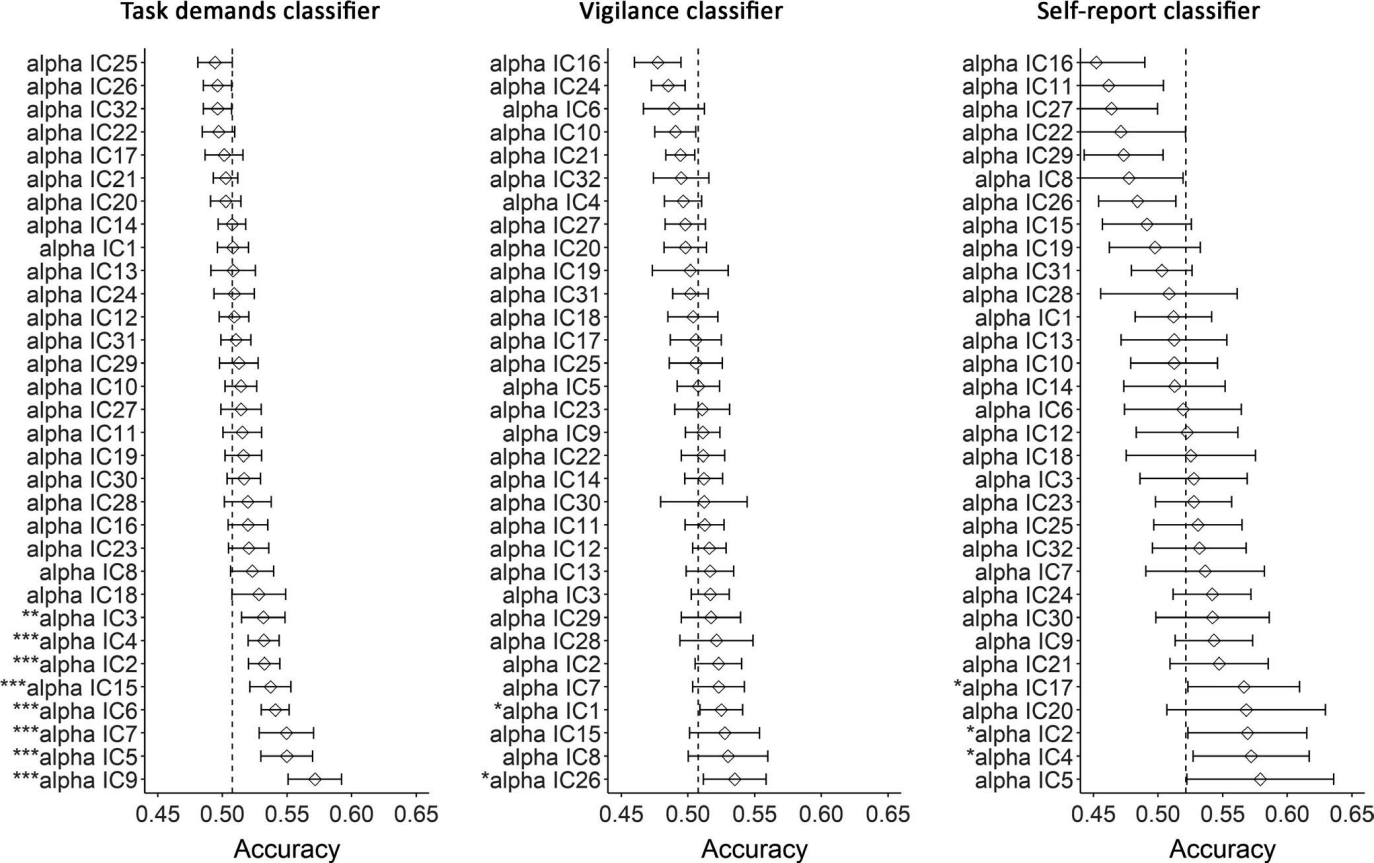
Feature testing results quantified as accuracy measured with LOPOCV separately for each independent component (IC) sorted by their mean accuracy. Accuracies that were significantly above‐chance level (vertical dashed line) are marked by asterisks before the corresponding IC label that also indicate the level of significance (*p < 0.05, **p < 0.01, ***p < 0.001). The error bar indicates 95% confidence interval

We then asked on what EEG features the classifiers based their predictions. The contribution of every feature to the classification was assessed by examining the accuracy of classification based on that single feature alone, computed through LOPOCV. These accuracies are shown in Figure 6. *t* tests comparing the prediction accuracy of each feature to chance level indicated that IC 2, 3, 4, 5, 6, 7, 9 and 15 were able to predict the level of task demands when assessed using LOPOCV. IC 1 and 26 could predict the participants' vigilance level when assessed using LOPOCV. IC 2, 4 and 17 could predict the participants' self‐reported mental state when assessed using LOPOCV. There were no ICs that could predict all three types of labels.

**FIGURE 7:**
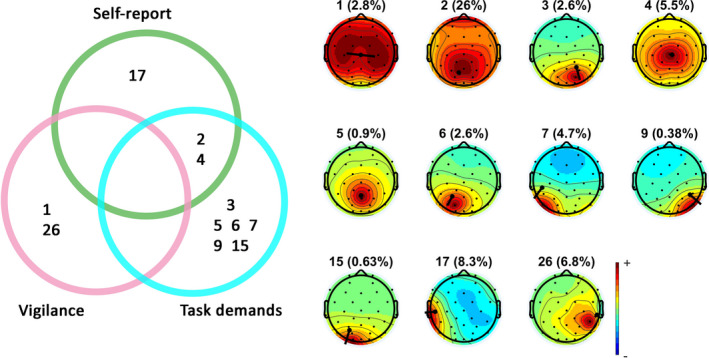
Left: Overlap in ICs used in prediction by the three classifiers. Right: Topoplots of the 11 significant ICs with dipole positions. The number in brackets indicates the residual variance (RV) associated with the respective component, essentially a measure of the error in the dipole fits [Colour figure can be viewed at wileyonlinelibrary.com]

Next, significant ICs were divided into groups based on whether their importance is restricted to one single classifier or whether it plays an important role in multiple classifiers. The results are shown in Figure 7 (left). We found IC 3, 5, 6, 7, 9 and 15 predicted only the level of task demands. IC 1 and 26 predicted only vigilance. IC 17 predicted only the self‐reported mental state. Besides, we found that IC 2 and 4 predicted both the self‐report and task demands. The scalp maps of all the significant ICs can be found in Figure 7 (right).

**FIGURE 8:**
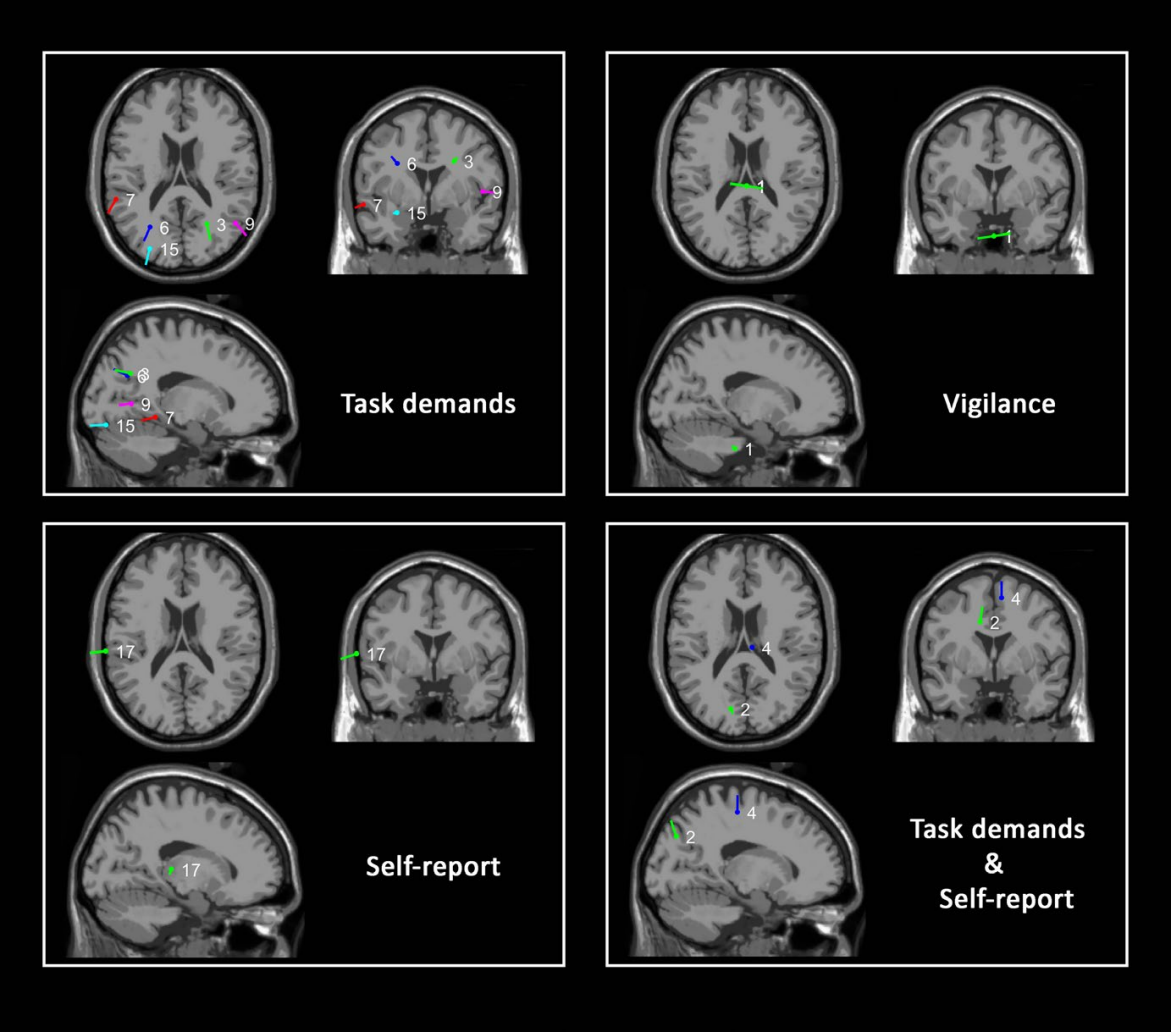
Source localization of the fitted dipoles of ICs that are significant exclusively in a single classifier and those that are significant in multiple classifiers [Colour figure can be viewed at wileyonlinelibrary.com]

A summary of the dipole‐fitting results of all the significant ICs can be found in Table 1. IC 1 had clear symmetric activity; thus, we fine‐tuned to fit two dipoles for this component following the recommended practice.^5^ After fitting IC1 with two dipoles, the residual variance (RV) dropped from 16% to the current 2.8%. The two fitted dipoles of IC1 overlapped in their coordinates, but their moments were oriented in different directions (Figure 7). The other components were all fitted with a single dipole. The dipole coordinates of IC 5 and 26 could not be matched to defined brain regions, and hence, those components were removed from further discussion. The mapping of the remaining dipoles to an MRI brain image can be found in Figure 8.

**TABLE 1:** MNI coordinates and brain area of the significant independent components (ICs)

IC	MNI‐*x*	MNI‐*y*	MNI‐*z*	Brain structure
1	0	−28	−38	Pons (brainstem)
2	−12	−77	35	Left precuneus
3	24	−62	30	Right precuneus
4	7	−23	56	Right medial frontal gyrus
5	0	−61	55	—
6	−27	−65	28	Left temporal lobe subgyral
7	−58	−40	−9	Left middle temporal gyrus
9	49	−61	3	Right middle temporal gyrus
15	−28	−84	−16	Left middle occipital gyrus
17	−68	−27	6	Left superior temporal gyrus
26	73	−37	16	—

Note

An empty place in the “area” column indicates a localization outside the defined brain regions. Note that IC1 was fitted to two dipoles with the same coordinate but different orientations.

**FIGURE 9:**
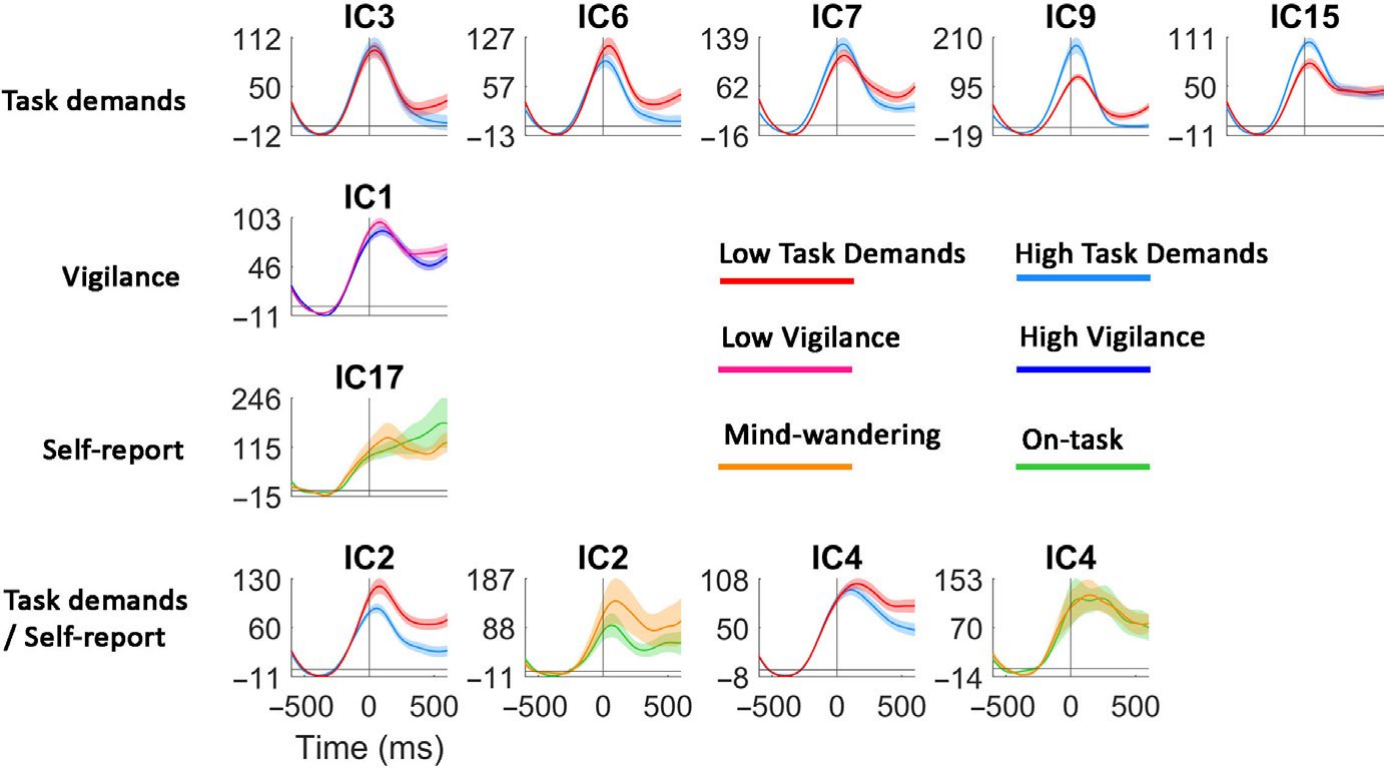
Time courses of IC differences in each categorization as a function of time [−600 600] millisecond relative to stimulus onset. Alpha power is indicated as a percentage change (%) compared to a [−600 –200] millisecond baseline. Note that baseline correction was used only for visualization purposes; during the machine learning, the raw power without baseline correction was used for feature computations. Plots of non‐baseline corrected alpha power can be found in Appendix S4. Shaded area indicates one between‐subject standard error (*SE*). The vertical and horizontal line indicates zero in both axes, respectively [Colour figure can be viewed at wileyonlinelibrary.com]

To understand how the ICs might differ between the two conditions within each categorization, we plotted the power of the significant ICs as a percentage change relative to a [−600 –200] millisecond baseline for both conditions in the respective categorizations in Figure 9.

## 4 DISCUSSION

The current research examined the hypothesis that low vigilance or performing a task with low demands is associated with a similar neural state as mind‐wandering. In order to test this hypothesis, we asked participants to perform a visual search task and a SART while measuring their momentary focusing state through intermittent probes. The analysis of the attentional rating scores to the probes indicated we were successful in manipulating participants' attentional state (Figure 4). Specifically, we found that participants reported a general decline in attention over the course of the task, as well as in a low‐ relative to high task demand situation. This rating result matched our assumption that low task demands or low‐vigilance levels would indicate a decrease in focus level.

However, despite the fact that behaviorally mind‐wandering, vigilance and task demands covaried, these factors were not associated with similar brain states (Figure 5) since—contrary to our expectation—neither a vigilance classifier nor a task demands classifier could predict the occurrence of mind‐wandering as measured by self‐report above‐chance level while a trained classifier trained specifically on self‐report could. This result is in contrast to both our hypotheses—that the neural correlate of mind‐wandering is equal to the neural correlate of low vigilance and of low task demands. The feature testing results (Figures 6 and 7) provided us with clues about where these mental states associated with vigilance, task demands and mind‐wandering may differ. A dipole‐fitting analysis of the significant ICs (Figures 8 and 9) indicated that the differences between mind‐wandering, low vigilance and task demands can be connected to specific neural correlates: Mind‐wandering is associated with the left superior temporal gyrus (STG, IC17); task demands are associated with the right precuneus (IC3), the left temporal lobe subgyral (IC6), the bilateral middle temporal gyrus (IC7, IC9), and the left middle occipital gyrus (IC15); vigilance is associated with the brainstem (IC1).

Despite the fact that we were in general not able to predict the occurrence of mind‐wandering by using classifiers trained on task demands or vigilance, we found that alpha power generated from the left precuneus (IC2) and the right medial frontal gyrus (IC4) was a common feature for both task demands and mind‐wandering.

In the remainder of this article, we will first discuss the observed neural distinctions between mind‐wandering and vigilance, followed by the similarities and differences between the neural correlates of mind‐wandering and task demands. We will end the discussion with limitations and future directions.

### 4.1 Mind‐wandering and vigilance decrement

We found alpha band power generated from dipoles located in the pons of the brainstem (IC1) to be a predictive feature of the vigilance classifiers. The brainstem is well known for its crucial role in regulating the cycle between waking and sleep (Buysse et al., 2004; Jones, 2011) and maintaining alertness through its connectivity to the thalamus (Mottaghy et al., 2006). Researchers further proposed a frontal–parietal–thalamic–brainstem network for internally motivated and controlled maintenance of alertness, and the right insula was found to be a key structure in that network (Clemens et al., 2011). While the current findings corroborate previous neuroscientific studies of alertness, we found IC1, which was localized in the brainstem, to have a stronger alpha desynchronization after stimulus onset in a high versus low‐vigilance state (Figure 9). To our knowledge, this link between brainstem alpha oscillation and vigilance has not been reported before.

For the self‐report classifiers, we found that alpha power generated from dipoles located in the left STG (IC17) was predictive only in this classifier. The left STG has been found to be an important neural correlate of semantic processing (Friederici, Rueschemeyer, Hahne, & Fiebach, 2003), social cognition (Grace, Rossell, Heinrichs, Kordsachia, & Labuschagne, 2018), and self‐related information processing (Qin et al., 2016). Moreover, the STG has been associated with memory recall (Mallow, Bernarding, Luchtmann, Bethmann, & Brechmann, 2015) and with problem‐solving (Tian et al., 2011). Together this suggests that the STG might help to organize and generate thinking content during mind‐wandering. We found that when mind‐wandering occurs, STG‐generated alpha band power desynchronized more after stimulus onset compared to when the person is in an on‐task state (Figure 9). Although reports about the alpha band activity on temporal sites are rare, there is one study examining intracranial EEG in the hippocampus, which suggests that alpha power decreases distinguished associative recognition from non‐associative item recognition (Staresina et al., 2016). This is somewhat consistent with our finding that alpha desynchronization in the STG might indicate a memory retrieval processes needed in the construction of daydreams.

To summarize, our study indicates that mind‐wandering and low‐level vigilance are distinctive phenomena, at least in their neural representations. While mind‐wandering requires the activity of the linguistic system and a memory retrieval‐associated neural structure (left STG), vigilance decrement is more about a general decline in the arousal system (brainstem), which manifests as reduced motivation and alertness. While reduced vigilance can give rise to mind‐wandering, our data suggest it does not reliably do so.

### 4.2 Mind‐wandering and task demands

Alpha power from several cortical areas was found to be predictive of task demands. Specifically, the areas that predicted task demands were the right precuneus (IC3), the left temporal lobe subgyral (IC6), the bilateral middle temporal gyrus (IC7, IC9) and the left middle occipital gyrus (IC15), forming a wide parietal–temporal–occipital network, spanning both hemispheres (see also Figure 8). This network has previously been suggested to be associated with general cognitive abilities (Ferreira et al., 2017), to facilitate the decoding of visual inputs and the preparation of motor processes (Lebar, Bernier, Guillaume, Mouchnino, & Blouin, 2015), and could be involved in visuoperceptual and visuospatial integration (Cerami et al., 2015). The current result that alpha power originating in multiple brain regions can predict task demands supports the above findings that the activity of the parietal–temporal–occipital network might functionally support the internal processes in performing a visual search task.

In this network, we note the involvement of the bilateral medial temporal gyrus (MTG). The MTG has been recognized as part of the DMN (Burkhouse et al., 2017), which is a distributed network of brain regions that is more active during rest than when performing a task (Raichle, 2015). Its deactivation has been correlated with improved attentional performance (Whitfield‐Gabrieli & Ford, 2012), and the DMN also plays a key role in generating daydreaming thoughts and mind‐wandering (Kucyi & Davis, 2014). Interestingly, the current evidence indicates the MTG could also be critically activated when performing tasks instead of mind‐wandering. This observation is consistent with the more recent view that the DMN is actively involved in task‐related mental activity as well as detailed representations of task relevant processes (Sormaz et al., 2018). For example, according to Turnbull et al. (2019), enhanced DMN‐occipital connectivity might be involved in maintaining task‐related details over time when the task required lower demands of visual processing. In line with this idea, the DMN has been found to be more decoupled from primary visual areas for participants who mind‐wandered more often in a reading task (Zhang, Savill, Margulies, Smallwood, & Jefferies, 2019). These examples also highlight the difficulty of detecting and classifying mind‐wandering: Many neural structures are involved both in on‐task and off‐task states.

We also found that the left precuneus (IC2) and the right medial frontal gyrus (IC4) are likely generators of alpha oscillations that were predictive in both the self‐report and the task demands classifiers. This finding is consistent with research that shows that the precuneus is involved in sensory processing (Clancy, Ding, Bernat, Schmidt, & Li, 2017), conscious visual perception (Bisenius, Trapp, Neumann, & Schroeter, 2015) and directing selective attention to goal‐related stimulus (Dosenbach et al., 2007). Interestingly, mind‐wandering has been proposed to be a sensory decoupling process (Schooler et al., 2011), in which cortical responses during sensory processing are reduced (Kam et al., 2011). In our research, the inhibited sensory processing was indicated by an enhanced alpha power in the precuneus (IC2). As shown in Figure 9, the effect was apparent through our experimental manipulation, in which the presented stimuli were kept consistent, and only the required mental effort could differ between the counting and the non‐counting tasks. In other words, reduced sensory processing could not be attributed to the external stimulus characteristics but only to an internal process.

The right medial frontal gyrus has been reported to be involved in inhibitory control (O'Brien et al., 2013). Previous work showed that deactivation of the medial frontal gyrus was associated with improved performance on a spatial working memory task (Liu et al., 2018). We found that alpha power in IC4 was higher in a low demand compared to a high‐demand condition, while not differentiating between the two self‐reported mental states (mind‐wandering vs. on‐task; Figure 9). This result seemed to match the behavior, in which we found a significant difference between task performance under different levels of demand, while no behavioral difference was found between the two self‐reported mental states.

To sum up, we found that the activity of a parietal–temporal–occipital network might be a neural marker of handling increasing task demands. We also found that the left precuneus and the right medial frontal gyrus might help to clarify the correlation between mind‐wandering and performing low‐demanding tasks.

### 4.3 Limitations and future work

Although we found that participants reported a general attentional decline in the second half of the experiment and the low‐demanding task as we expected, the average rating scores of attentional states were relatively high. The mean mind‐wandering scores for both the second half (low vigilance) and the low task demands was nearly zero, and rarely negative, which would have indicated mind‐wandering (Figure 4). This indicated that participants may have been influenced by the phrasing of the question “To what degree were you focusing on the task just now?”—nudging them to report in the “focused” direction when rating on the scale. A similar effect was reported before, as asking for “mind‐wandering” rather than for “focused” would increase participant's reports of mind‐wandering (Weinstein, De Lima, & van der Zee, 2018). Although we also presented “mind‐wandering” in the rating scale as the label for the negative values, we cannot rule out the possibility that the reports were framed by the probe.

Individual participants varied strongly in their attentional fluctuations. Some participants kept a relatively strong task focus during the experiment, which was indicated by a higher rating score, while some others had attentional drift during the experiment, so that their reports included an equal amount of negative and positive values. Since one of the current machine‐learning goals was to test the classifiers in the left‐out participant, not only those data with varied mental fluctuation were included, but also those with a relatively stable attentional state (e.g., always focused), so that the predictivity of the classifier was tested on a quite heterogeneous data sample. This might have limited our prediction performance. It might be interesting to explore in the future if keeping the data sample relatively homogenous, for example, training and testing the classifiers among the data of the participants who reported large attentional fluctuations, could improve the prediction accuracy in general.

The third limitation is that the current analysis was performed on a relatively narrow time window around the stimulus. It is likely that the mental state changes (e.g., on‐task shifts to mind‐wandering) happen later after the stimulus onset, not necessarily time‐locked to visual stimuli. In the current study, to rule out the possibility that the classifiers used motor‐related potentials or artifacts to classify, we decided to use a short time window. It would be interesting for future research to expand the time window used for classification and thereby to explore this more dynamic view of mind‐wandering.

One special note here is that the objective of our current research was not to obtain maximal accuracy in predicting mind‐wandering (or any of the other experimental manipulations, e.g., vigilance, task demands). The goal of the current research is to compare three classifier's performance in predicting mind‐wandering, to examine whether vigilance decrement/low task demands could predict mind‐wandering, instead of achieving the optimal classification performance. As long as the classifiers performed above‐chance level during the LOPOCV and 10‐fold CV, we regarded the classifiers as valid. Meanwhile, when predicting mind‐wandering, the vigilance/task demands classifier performed worse than the self‐report classifier, leading us to reject our hypothesis that “vigilance decrement/ low task demands == mind‐wandering.” Nevertheless, this does not mean that better performance of classifiers is not possible with more complex classifiers, other features or larger datasets. For example, Zanesco, Denkova, Witkin, and Jha (2019) used Markov chain modeling to reveal the hidden states underlying time series of probe ratings (mind‐wandering rating scale similar to the current study). It would be interesting to explore if such models can be combined with EEG to reveal the temporal dynamics of the changing mental states.

Future work could also consider using other dimensions of thoughts such as the levels of detail, the modality or the emotional valence of the thoughts to classify the thought content in relating it to brain activity (Ho et al., 2019). For example, the connectivity pattern within the DMN has been found to discriminate between levels of detail of ongoing thought (Sormaz et al., 2018) and the medial orbitofrontal cortex (mOFC) has been shown to code for the affective tone of the thought (Tusche, Smallwood, Bernhardt, & Singer, 2014). Exploring the neural correlates of these features could further help to understand the generation and prevalence of different kinds of mind‐wandering.

## 5 CONCLUSION

Our study has shown that mind‐wandering is a different mental phenomenon from both low vigilance and being in a low‐demand task situation, because each of these processes had unique neural correlates. Mind‐wandering is associated with activity arising from the left STG, possibly reflecting the thought generating process. Low vigilance is characterized by alpha oscillations arising from the brainstem. Low task demands are associated with activity in a parietal–temporal–occipital network. Mind‐wandering and performing a low‐demanding task both activate the left precuneus and the right medial frontal gyrus, possibly reflecting a process of sensory decoupling. More generally, this study demonstrates how machine‐learning classifiers can help to unveil similarities and differences between mind‐wandering and other mental processes by comparing their characteristic neural markers.

## CONFLICT OF INTEREST

There was no conflict of interest with respect to the publication or authorship of this article.

### Peer review

The peer review history for this article is available at https://publons.com/publon/10.1111/ejn.14863

## AUTHOR CONTRIBUTIONS

Christina Y. Jin involved in conceptualization, methodology, software, formal analysis, investigation, writing—original draft, and visualization. Jelmer P. Borst and Marieke K. van Vugt involved in conceptualization, methodology, resources, writing—review and editing, and supervision.

## Supporting information

Appendix S1‐S4

## Data Availability

The data that support the findings of this study are available from the corresponding author upon reasonable request.

## Footnotes

^1^ The window length was based on previous work predicting mind‐wandering in EEG (Jin et al., 2019), in which we tested the difference in the classification results when using data from 30 and 15 s before each probe question. In that study, we found that the classifier trained using the shorter time window (15 s) did not outperform the one trained using the longer time window (30 s). We therefore decided to use a 24‐s time window before each probe in the current study. Another consideration is that shrinking the time window would cut the training data sample size, thereby potentially causing trouble for the machine‐learning procedure.

^2^ The log‐transformed RTs of each group for *t* tests were examined by the Shapiro–Wilk tests of normality. None of the null hypothesis that the population is normally distributed was rejected for an alpha level of 0.05.

^3^ We used the interval up to 600 ms after the stimulus because that was close to the time when participants finished making a response in the SART (mean response time is around 500 ms as shown in Figure 3). In the visual search task, participants were still processing the stimulus at this time because in this task participants were required to wait with responding until the search panel disappeared. After participants finished a response, their cognitive state and motor actions would be difficult to control. Therefore, we decided to cut the window at 600 ms after the stimulus onset. To make a symmetric time window, we also took 600 ms before the stimulus onset into consideration because pre‐stimulus activity may be predictive of our variables of interest as well.

^4^ Because we trained the task demands and vigilance classifiers over all trials while the self‐report classifier was built upon three trials before each probe, to rule out the possibility that the higher performance in the self‐report classifier in prediction the self‐reported mental states in the SART could be caused by a lower amount of data, we performed the same analysis with all classifiers trained with three trials before each probe. The results can be found in Appendix S1. Just as the results in the main text, both the task demands and vigilance classifiers performed above the chance level during the 10‐fold CV and LOPOCV (*t*s > 2.08, *p*s < 0.04). When predicting the self‐reported mental state in both tasks, the task demands and the vigilance classifier did not surpass chance level (*t*s < 0.37, *p*s > 0.164).

^5^
https://sccn.ucsd.edu/wiki/A08:_DIPFIT
