# Supplementary material for: Distinguishing vigilance decrement and low task demands from mind‐wandering: A machine learning analysis of EEG
Source: Eur J Neurosci. 2020 Jun 28;52(9):4147–64. doi: 10.1111/ejn.14863 (PMC7689771; doi:10.1111/ejn.14863)
Supplement: Supplementary file 1 — Appendix S1‐S4 [file EJN-52-4147-s001.pdf]

Appendix S1

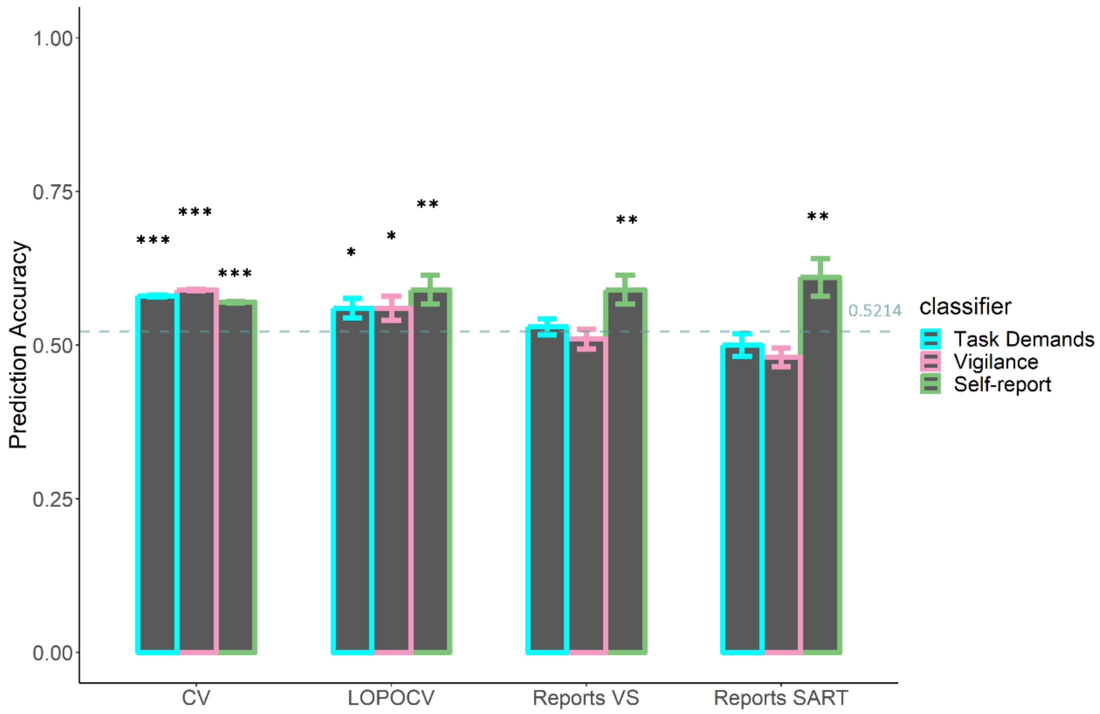

Figure A. Performance with classifiers trained on three trials before each probe. This graph is the result of a supplementary analysis as to the main results showed in Figure 5. All the classifiers performed above the chance level (0.5214) during the 10-fold CV and LOPOCV ($ts > 2.08, ps < 0.046$). When predicting the Self-reported mental states in both tasks, the Task demands or the Vigilance classifier did not surpass the chance level ($ts < .37, ps > 0.164$). The error bar reflects one between-subject standard error (SE). Asterisks indicate the difference between the accuracy and chancel level is significant using one-sample t-tests (*** p <0.001, ** p<0.01).

Appendix S2

Trial count in each condition for each participant during the behavioral analysis (compensatory data to section 3.1). Shaded areas indicate that a participant was removed from the behavioral analysis in the corresponding task due to the small amount of data in one of the two classes. Note that this procedure was only performed during the behavioral analysis. Because in the machine learning analysis we trained classifiers on 29 participants and tested them on the remaining left-out participant (LOPOCV), this "missing one class" dilemma is not a problem since the training data sample is guaranteed to have data from two classes and the test sample was allowed to be of data from a single class.

	Visual Search Task		SART	
Participant	On-task	Mind-wandering	On-task	Mind-wandering
1	21	30	36	0
2	15	45	33	3
3	54	3	27	9
4	60	0	36	0
5	15	45	9	27
6	57	0	36	0
7	24	15	33	0
8	51	9	36	0
9	60	0	36	0
10	15	42	6	27
11	24	33	9	12
12	36	24	36	0
13	51	0	36	0
14	57	3	36	0
15	30	30	21	6
16	42	15	30	0
17	39	15	9	24
18	12	24	15	15
19	39	21	30	3
20	54	3	36	0
21	15	33	6	30
22	51	6	36	0
23	18	39	24	6
24	3	57	0	36
25	39	15	24	6
26	54	0	36	0
27	42	9	36	0
28	33	12	27	3
29	24	24	9	12
30	30	6	18	0

Appendix S3

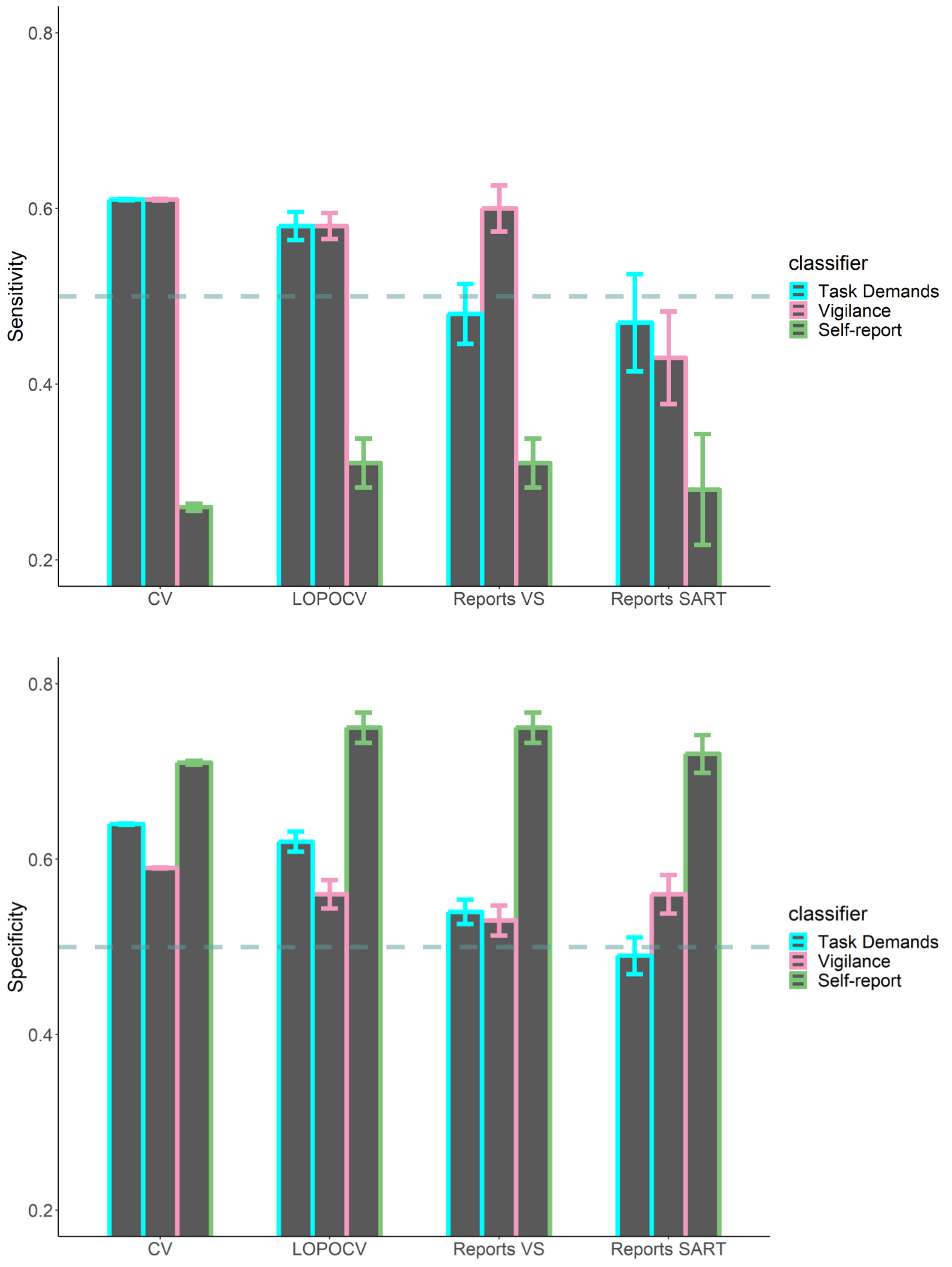

Figure B. Sensitivity and specificity as indications of biased detection of each classifier compensatory to the achieved accuracy in Figure 5. Sensitivity is the true positive rate, which is the ratio of the correctly classified positive cases among all the positive cases. Specificity is

the true negative rate, which is the ratio of the correctly classified negative cases among all the negative cases. In the current study, positive cases are as “low demands”, “low vigilance” or “mind-wandering”, and negative cases are defined as “high demands”, “high vigilance” or “on-task”.

Appendix S4

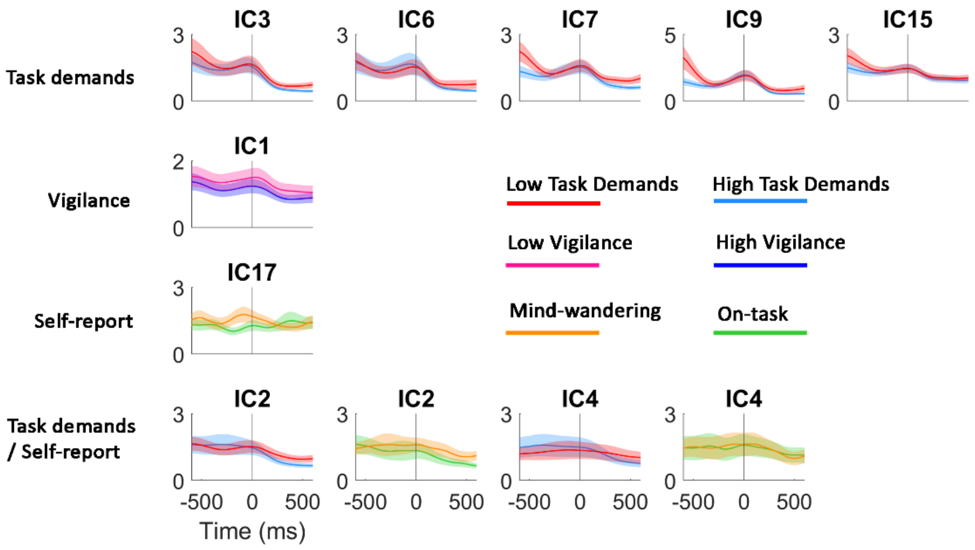

Figure C. The same features in Figure 9 plotted without baseline correction. This figure shows that in general, alpha power suppressed after stimulus-onset.
